# Irradiance uniformity optimization for a photodynamic therapy treatment device with 3D scanner

**DOI:** 10.1117/1.JBO.26.7.078001

**Published:** 2021-07-15

**Authors:** Xu Wang, Wen-Rui Kang, Xiao-Ming Hu, Qin Li

**Affiliations:** Ministry of Industry and Information Technology, Beijing Institute of Technology, School of Life Science, Key Laboratory of Convergence Medical Engineering System and Healthcare Technology, Beijing, China

**Keywords:** photodynamic therapy, light source optimization, skin disease

## Abstract

**Significance:** The light dose in photodynamic therapy (PDT) has a considerable influence on its treatment effect, and irradiance uniformity is an issue of much concern for researchers. However, achieving intelligent and personalized dosimetry adjustments remains a challenge for current PDT instruments.

**Aim:** To meet the requirements of intelligent and personalized dosimetry adjustments for the light dose on an irregular surface, a new PDT device with its optimal control method is proposed.

**Approach:** This research introduces a new PDT device that includes a 3D scanner, a light-emitting diode (LED) array, and a computer. The 3D scanner is proposed to generate the point cloud of the lesion and the LED array light source, and obtain the relative position and rotation parameters between them. Then, an image segmentation algorithm is used to segment the lesion point cloud into several cluster regions. Last, the current of each LED unit is adjusted separately to achieve the expected irradiance on each cluster.

**Results:** Compared with the general light source, the optimized light source increases the effective irradiance area by 9% to 15% and improves its uniformity by ∼9% on a human port-wine stain head model.

**Conclusions:** The device and its optimal method may be used for optimizing the light dosimetry to realize intelligent and personalized treatment.

## 1 Introduction

Photodynamic therapy (PDT) is a new method of treating clinical diseases in recent years, especially in dermatology.^1^ PDT is a dynamic interaction involving a photosensitizer, oxygen, and light. A photosensitizer is injected into the human body and retained in the lesion tissue. After a specific wavelength of light irradiance is received, a photochemical reaction occurs between the photosensitizer and the oxygen in the tissue, and reactive oxygen with cytotoxicity is also generated; this destroys the lesion tissue and achieves the therapeutic purpose.^2^^–^^4^

The clinical efficacy of PDT depends partially on the light dose delivered to the target area.^5^^–^^8^ Studies have shown that overdosing has a side effect with the risk of scarring, but a slight deficiency in the light dose may fail to achieve the expected clinical outcomes.^9^^–^^11^ Such a failure causes undue stress and time for the patient and the healthcare system. However, due to individual differences, accurately adjusting the irradiance according to the shape and size of the lesion on an irregular surface is necessary to avoid damage to surrounding normal tissues and optimize the treatment, by adjusting the light dose according to the degree of local lesions.^12^ Therefore, the light dose, including irradiance ($\mathrm{mW}/{\mathrm{cm}}^{2}$) and radiance ($J/{\mathrm{cm}}^{2}$), is one of the most important factors during PDT, which is a highly localized treatment.^13^^,^^14^ In practice, most clinical PDT researchers worldwide pay attention to the calculation or measurement of the delivered light dose on a lesion surface. Beigzadeh et al.^14^ proposed a method of measuring the light dose in water, which is generally considered the best equivalent medium for human tissue. This method is based on digital holography and has the advantage of providing continuous optical dose distribution for phantom tissue. Kim et al. developed a PDT dosimeter that simultaneously measures the concentration of the light dose and a photosensitizer through eight isotropic detectors to collect data on the light dose in the cavity. To track the position of the light source in the treatment cavity during light delivery, an optical infrared navigation system was designed to monitor the reflection passive mark on the modified and improved treatment transfer rod.^15^^,^^16^

Many other factors must be considered when choosing the right light source. Two of the most important factors are its irradiance ($\mathrm{mW}/{\mathrm{cm}}^{2}$) and homogeneity. The radiance ($J/{\mathrm{cm}}^{2}$) should be high enough to provide a good therapeutic response for thick, dark, and deep lesions, whereas the irradiance must be low enough to reduce pain and avoid scar formation for children and lightly colored superficial lesions.^17^^,^^18^ A light-emitting diode (LED) has been successfully applied in the treatment of many skin diseases in PDT due to its high cover area and uniformity.^19^^–^^22^ Other light modulators, such as a micromirror, are also used for PDT to achieve homogeneous irradiance by modulating the pulse duty factor. However, the coupling factor of such a device is limited by a digital mirror device.^23^ Another medical manipulator system with a binocular vision system was also built to supervise the PDT operation, but the uniformity of laser irradiance was not discussed.^18^ Therefore, a new LED array treatment device and its light dose uniformity optimization method for PDT is proposed in this research. According to the properties of a light source and the spatial relationship between a lesion and a light source, the light intensity matrix of LED can be solved. The light intensity of each LED can also be adjusted according to the calculated matrix value to allow every patch of the surface lesion to receive the expected irradiance.

## 2 Materials and Methods

### 2.1 System Composition

**Fig. 1:**
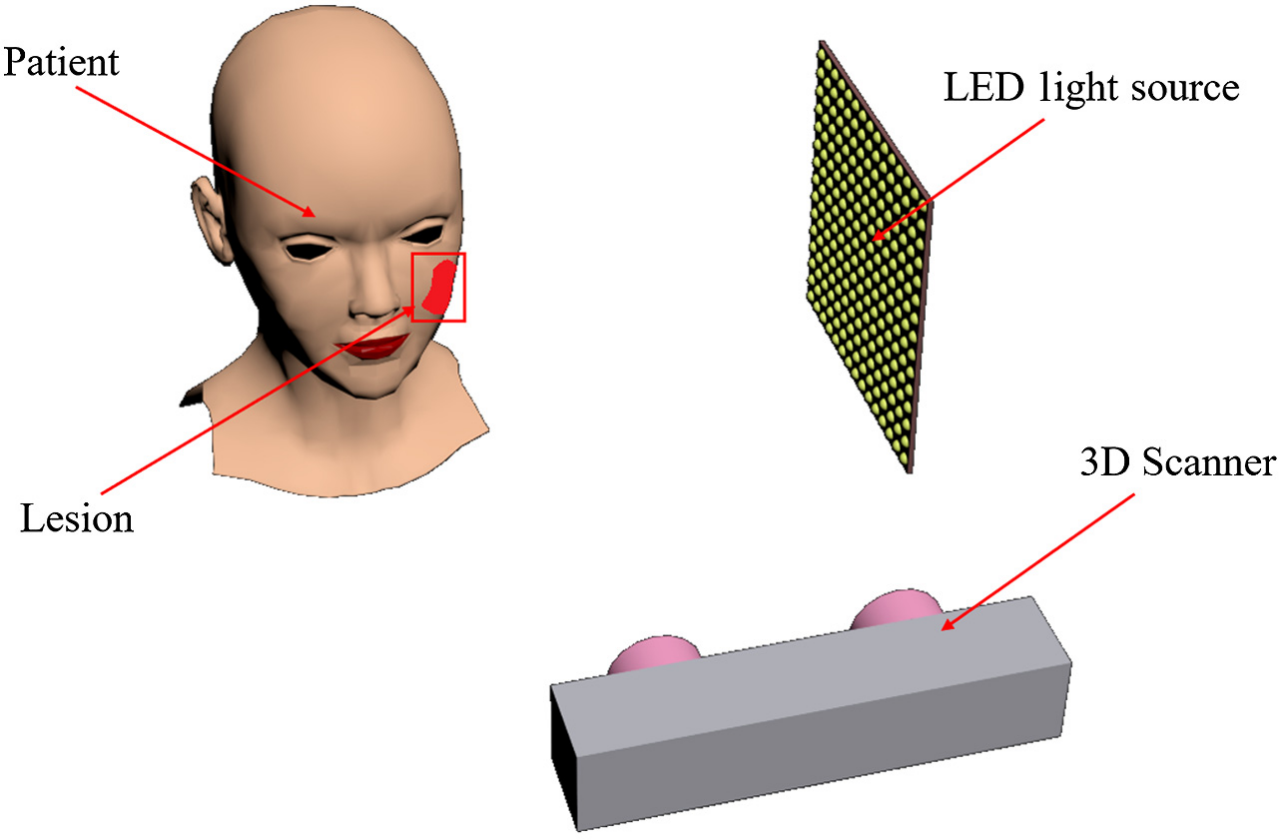
System composition diagram.

The proposed demo system (Fig. 1) comprises a 3D scanner, an LED-array panel, and a head model with skin lesions. The point cloud data of the head model are obtained with an industrial 3D scanner (Surface120, Zhixiang, China) with a resolution of depth map of 640×400, resolution of color image of 2560×1600, and field of view of 52  deg×31  deg. The point accuracy of the 3D scanner is ±0.22  mm at a distance of 50 cm. The LED-array panel comprises 15 rows and 15 columns of LEDs with an interval of 1 cm. The specifications of each LED (XLamp^®^ XQ-E, Cree) are the same, and the current can be adjusted independently from 0 to 1 A with an irradiance intensity up to 750  mW/Sr. The lens (FP16558_LISA3-RS-PIN, Ledil, Finland) couple efficiency is 90%, with a half angle of 15 deg. The head model is purchased from a market, whereas the skin lesions are painted red to model the port-wine stains.

### 2.2 Surface Topography from Point Cloud Acquisition

According to the pinhole linear model, the 3D point cloud can be projected first onto a two-dimensional (2D) plane with a determined mapping relationship. The 2D image can then be converted from RGB color space to CIE-L*a*b color space. The brightness channel (L channel) is normalized to eliminate the influence of uneven illumination on lesion extraction. Subsequently, the foreground and background saliency of pixels are constructed using the color difference between the skin lesions and the foreground and background eigenvalues. The pixels are classified according to saliency by threshold segmentation. Finally, the 2D image of the lesion is segmented, and the 3D point cloud of the lesion is obtained through the mapping relationship between the 3D point cloud and the 2D image.^24^^,^^25^

For our head model, the point cloud data of lesions generally have thousands of points, and the illumination range of each LED unit covers hundreds of points. In general, three points make a triangle patch. The whole lesion is divided into dozens of small cluster patches. The weighted center of mass distance method is used to aggregate a hierarchical cluster tree for all points. A maximum of n clusters with the distance criterion is also constructed. Thus, the calculation and optimization for the driving current of each LED is simplified by setting the expected irradiance on the clustering center points.

### 2.3 Light Intensity Adjustment

According to the properties of a light source and the spatial relationship between a lesion and a light source, the light intensity of each LED is calculated for a target irradiance value on the lesion. The irradiance of a certain point on the lesion from a single LED source is expressed as follows:^26^

$$E_{p}=\frac{I_{\left(\varphi \right)}\cdot \cos(\theta )}{r^{2}},$$ (1)

where $I_{\left(\varphi \right)}$ is the radiation intensity of a single LED, θ is the angle between the normal vector of point cloud and the connection vector from the center of the LED to the point, and r is the distance from this point to an LED. The intensity distribution of LED is approximated by Eq. (2) as follows:

$$I_{\left(\varphi \right)}=I_{0}\cdot \cos^{m}(\varphi ),$$ (2)

where $I_{0}$ represents the light intensity on the direction of normal vector and φ is the angle between the LED normal vector and the connection vector between the LED to a point. The coefficient m is calculated as

$$m=\frac{-\ln\;2}{\ln(\cos(\psi ))},$$ (3)

where ψ is the half luminous angle. Thus, the expected irradiance of the illumination point from a single LED corresponds to

$$E_{p}=I_{0}\cdot \frac{\cos(\theta )\cdot \cos^{m}(\varphi )}{r^{2}}.$$ (4)

Under the illumination of an array light source, the irradiance of a certain point of the lesion should be the sum of the irradiance of all LED units at that point:

$$E_{T}=\overset{225}{\underset{i=1}{\sum }}I_{0}^{\left(i\right)}\cdot \frac{\cos(\theta_{i})\cdot \cos^{m_{i}}(\varphi_{i})}{r_{i}^{2}}.$$ (5)

Then, it is transformed into solving the least square curve fitting problem, which minimizes the difference between the actual irradiance and the expected irradiance of each cluster center as follows:

$$\underset{I_{0}}{\min}\;\frac{1}{2}{\left\|E_{T}-\overset{˜}{E}\right\|}_{2}^{2},$$ (6)

where the solution range of $I_{0}$ is $ls\leq I_{0}\leq hs$ with ls and hs being the lower and upper limits of the light intensity of each LED, respectively, that are modulated by its driver current and $\overset{˜}{E}$ is the expected irradiance on the surface of skin lesions.

## 3 Results

### 3.1 Irradiance Distribution on a Typical Surface

**Fig. 2:**
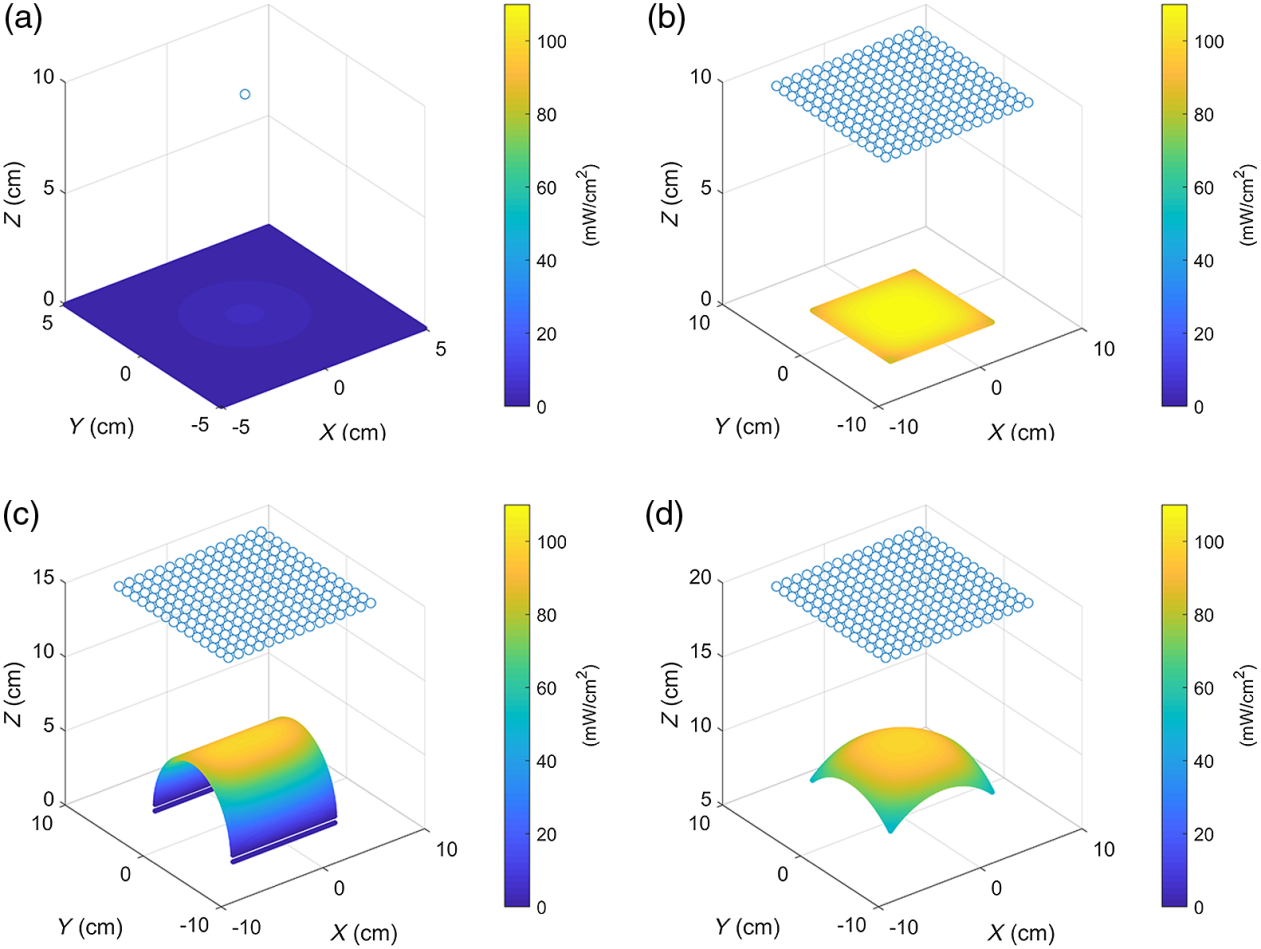
Distribution of irradiance on a typical surface: (a) single LED on a plane and LED array on (b) a plane, (c) a cylinder, and (d) a sphere.

The ability of the proposed LED array panel to form effective irradiance on a typical lesion surface was verified. Figure 2 shows the irradiance distribution map of a single LED and a 15×15 LED array on a plane, a cylindrical surface with a radius of 5 cm and a height of 10 cm, and a sphere with a radius of 10 cm. The verification area is on the XOY plane (parallel to the plane of the LED array) within a square of 10  cm×10  cm. Taking the irradiance requirement for port-wine stain PDT as an example, the expected irradiance on the surface of skin lesions is $100(\pm 10)\;\mathrm{mW}/{\mathrm{cm}}^{2}$. When the light intensity of a traditional LED light source is at 372  mW/Sr, the maximum irradiance on the plane surface at a distance of 10 cm reaches $110\;\mathrm{mW}/{\mathrm{cm}}^{2}$ [Fig. 2(b)]. The coefficient of variation and effective irradiance occupancy rate of these surface types under the LED array panel are calculated and listed in Table 1, where the coefficient of variation refers to the quotient between the standard deviation and the average of the irradiance of all points on the surface. Given that the point cloud is evenly distributed in the lesion cloud, the evaluation index of effective proportion is the ratio between the number of points in which the irradiance on it is within the range of the expected irradiance and the total number.

**Table 1:** Statistics on a typical surface.

	Total number of points	Effective proportion (%)	Coefficient of variation (%)
Plane	34,596	85.63	5.11
Cylinder	18,416	45.58	36.16
Sphere	25,848	63.98	12.28

### 3.2 Image Acquisition and Point Cloud Cluster

**Fig. 3.**
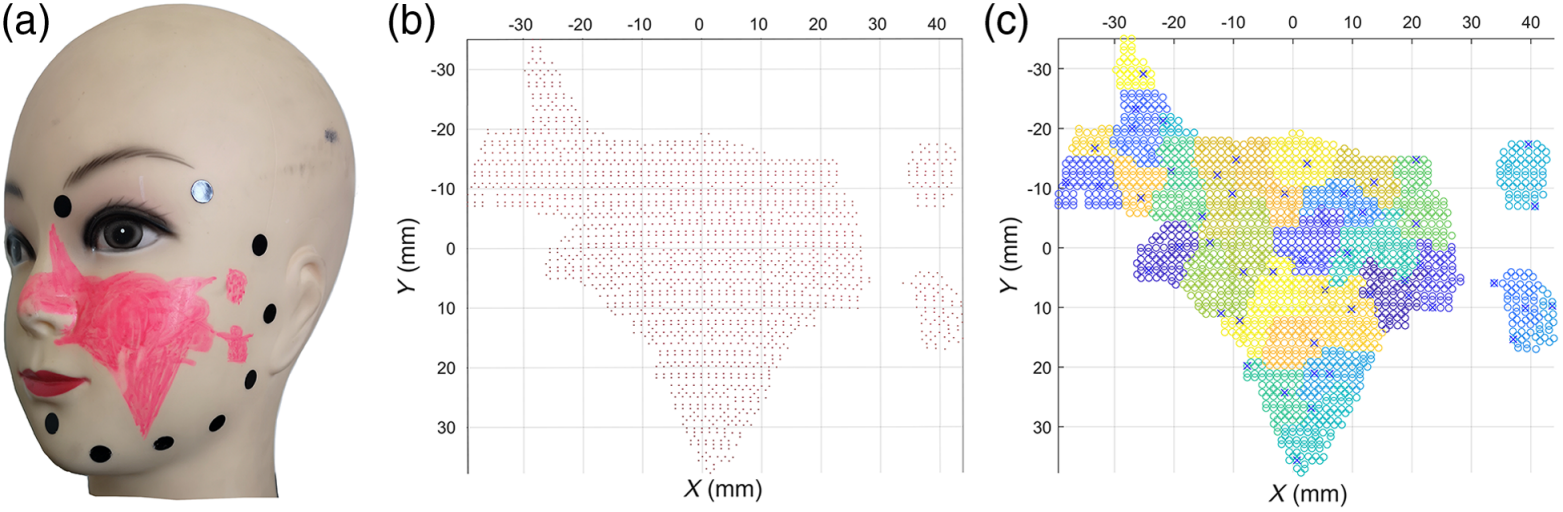
Head model point cloud segmentation and clustering: (a) head model, (b) segmented lesion point cloud, and (c) point cloud clustering.

In our test, the number of point clouds for the head model is 50,754, and the average distance between points is 0.69 mm. The segmented lesion cloud is composed of 1941 points, as shown in Fig. 3(b). The lesions are divided into 50 regions by point cloud clustering technology, as shown in Fig. 3(c). Different clusters are rendered by a pseudocolor map in which the circle represents all points in the point cloud and the cross represents the cluster center.

### 3.3 Light Intensity Matrix

**Fig. 4:**
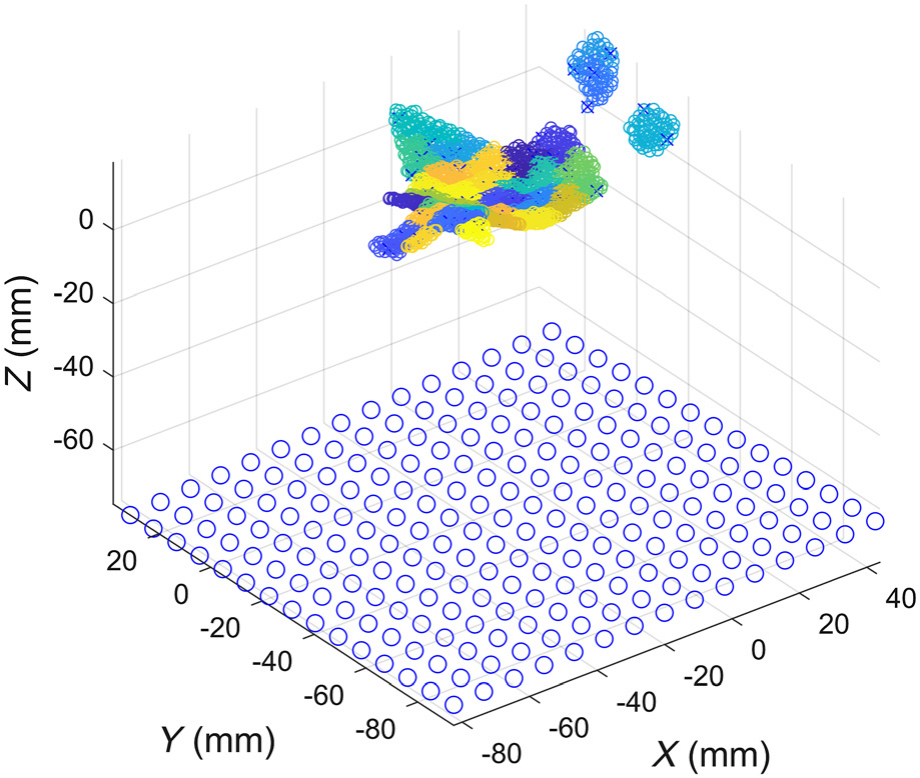
Diagram of the relationship between an LED array and a lesion.

Three adjacent points form a patch, and the normal vector set of the patch is used to replace the normal vector set of the point cloud. The normal vector of the light source plane is collinear with the average normal vector of the lesion point cloud. The light source is placed 10 cm away along the average normal vector of the lesion cloud (Fig. 4).

**Fig. 5:**
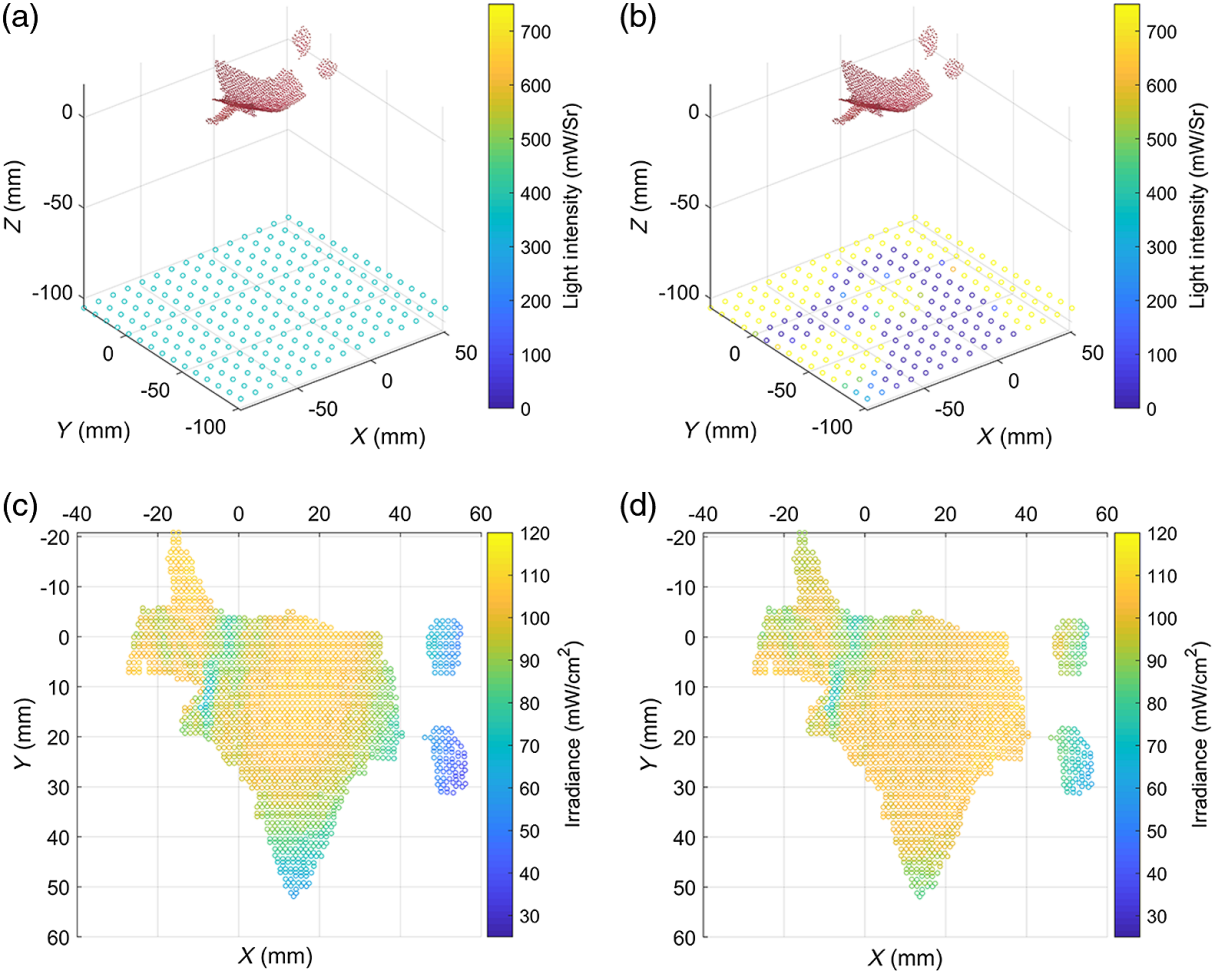
The light source intensity and irradiance distribution before and after optimization. The light intensity for (a) general operation and (b) after optimization and the irradiance distribution on lesion for (c) the general operation and (d) after optimization.

Each cluster region on the lesion is expected to reach $100\;\mathrm{mW}/{\mathrm{cm}}^{2}$ but not more than $110\;\mathrm{mW}/{\mathrm{cm}}^{2}$. The traditional light source with constant 372  mW/Sr diode light intensity is used to irradiate the lesion as the control group. The $E_{T}$ value in Eq. (6) ($100\;\mathrm{mW}/{\mathrm{cm}}^{2}$) is used in our approach as the experimental group, considering the pulse width modulation ability of the LED driver. The solution range of $I_{0}$ within 0 to 750 mW/Sr is regarded as the constrained condition to compare the performance between the general and optimized operations. The intensity value and the irradiance distribution of the lesion surface are shown in Fig. 5.

**Fig. 6:**
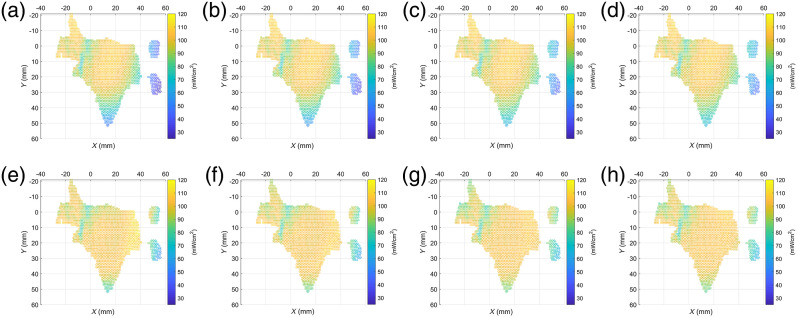
Irradiance distribution (a)–(d) without and (e)–(h) with optimization at different distances: (a) 80 mm, (b) 90 mm, (c) 110 mm, (d) 120 mm, (e) 80 mm, (f) 90 mm, (g) 110 mm, and (h) 120 mm.

### 3.4 Statistics and Evaluation

The irradiance on every point in the point cloud before and after the optimization of $I_{0}$ of the LED is analyzed in Fig. 6, and the distances of the LED and the point cloud are 80, 90, 110, and 120 mm. In the general group, the distance between 110 and 120 mm is too large to achieve an effective irradiance. Therefore, under the premise of the maximum effective irradiance not exceeding $110\;\mathrm{mW}/{\mathrm{cm}}^{2}$, the normal light intensity of the LED was increased to 411 and 449  mW/Sr in both experiments. However, the distance between 80 and 90 mm was too short. Thus, the maximum effective irradiance is greater than $110\;\mathrm{mW}/{\mathrm{cm}}^{2}$. Moreover, the normal light intensity of LED is set to 296 and 334 mW/Sr in the two experiments. The irradiance distributions before and after optimization are shown.

**Fig. 7.**
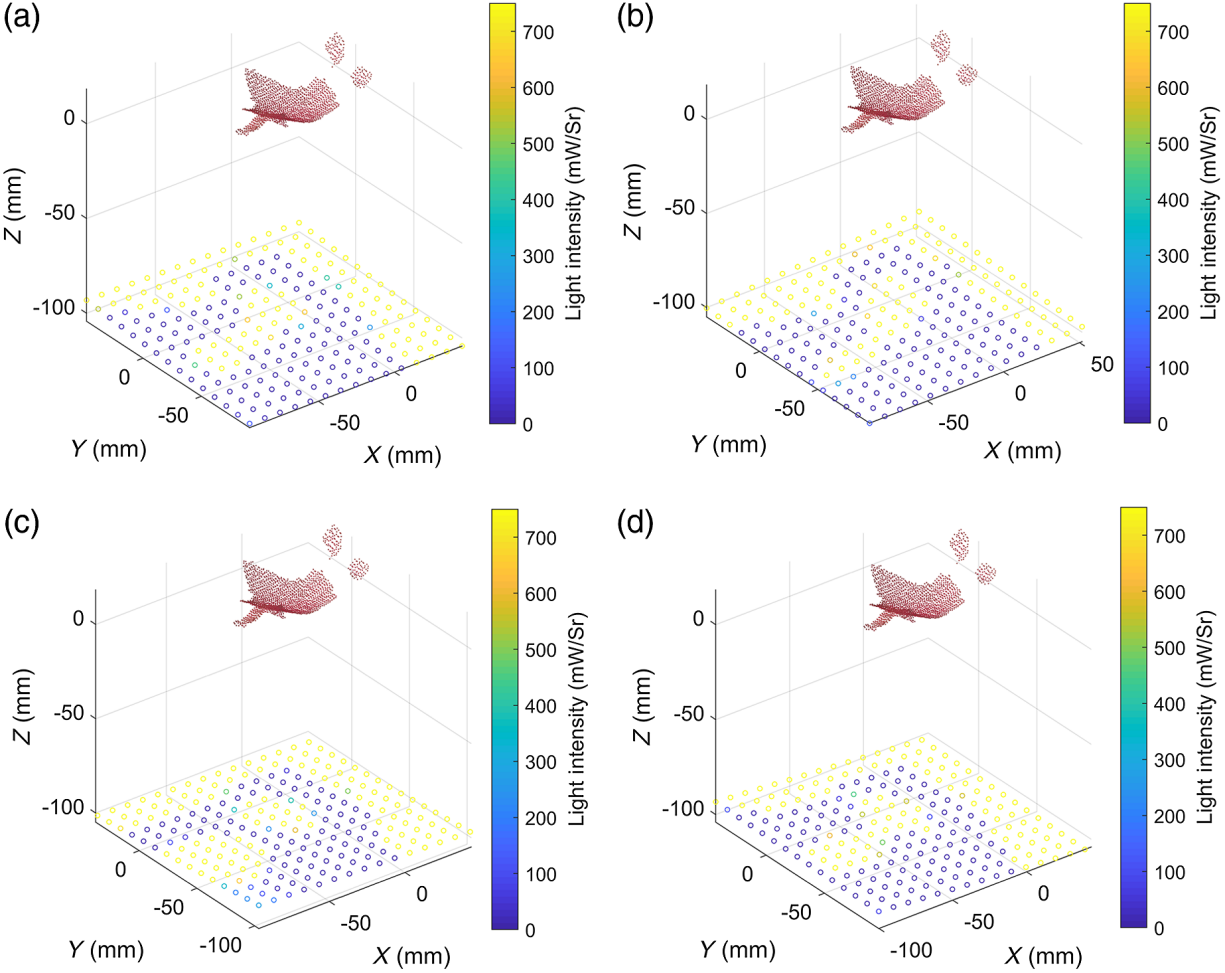
Optimized light-source illumination at different angles: (a) −10  deg, (b) −5  deg, (c) 5 deg, and (d) 10 deg.

**Fig. 8:**
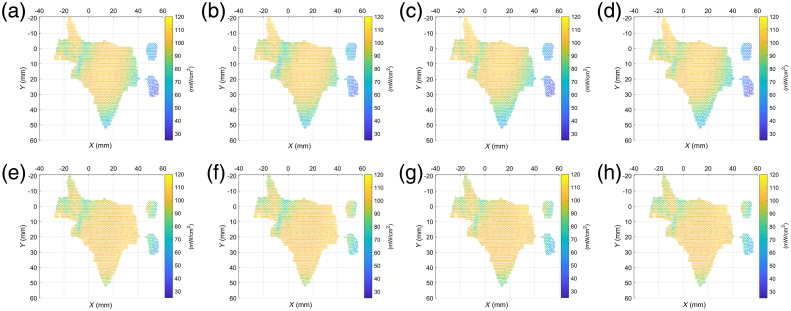
Irradiance distribution (a)–(d) without and (e)–(h) with optimization at different angles: (a) −10  deg, (b) −5  deg, (c) 5 deg, (d) 10 deg, (e) −10  deg, (f) −5  deg, (g) 5 deg, and (h) 10 deg.

The effects of rotation on the stability of our method are also evaluated. Taking the center of the lesion as the origin, the center of gravity of the light source as the vertex, and the distances between the center of the LED panel and the point cloud at 110 mm, the normal vector of the light source plane is rotated around the unit vector (0, 0, 1) by various angles (−10  deg, −5  deg, 5 deg, and 10 deg) for verification, as shown in Fig. 7. Meanwhile, the irradiance distributions before and after optimization are shown in Fig. 8.

Intuitively, the irradiance distribution of the optimized light source on the lesion is generally more uniform than that of the general light source. The uniformity is tested accurately by determining the effective irradiance and coefficient of variation of all points through the adjustment of the relative angle and the distance between the LED array and the lesion, as shown in Table 2.

**Table 2:** Evaluation of irradiance distribution before and after optimization.

		Before optimization			After optimization		
Distance (mm)	Angle (deg)	Number of points	Proportion (%)	Coefficient of variation (%)	Number of points	Proportion (%)	Coefficient of variation (%)
120	0	1458	75.23	14.08	1642	84.73	8.60
110	0	1441	74.36	14.83	1642	84.73	8.43
100	0	1422	73.37	15.84	1694	87.41	8.26
90	0	1398	72.14	17.23	1671	86.22	8.75
80	0	1372	70.80	19.22	1597	82.40	9.64
100	−10	1403	72.39	15.64	1666	85.96	8.50
100	−5	1451	74.87	14.96	1674	86.34	8.07
100	5	1399	72.19	17.10	1667	86.02	8.95
100	10	1384	71.41	16.92	1676	86.48	9.10

According to the statistical results, the proportion of the effective irradiance area of the optimized light source to the lesion model is increased by 9% to 15%. In addition, the variation coefficient of the irradiance on the lesion point cloud indicated that the uniformity of the optimal method is better than that of the general device.

## 4 Discussion

### 4.1 Correction Ability for Typical Surface

**Fig. 9:**
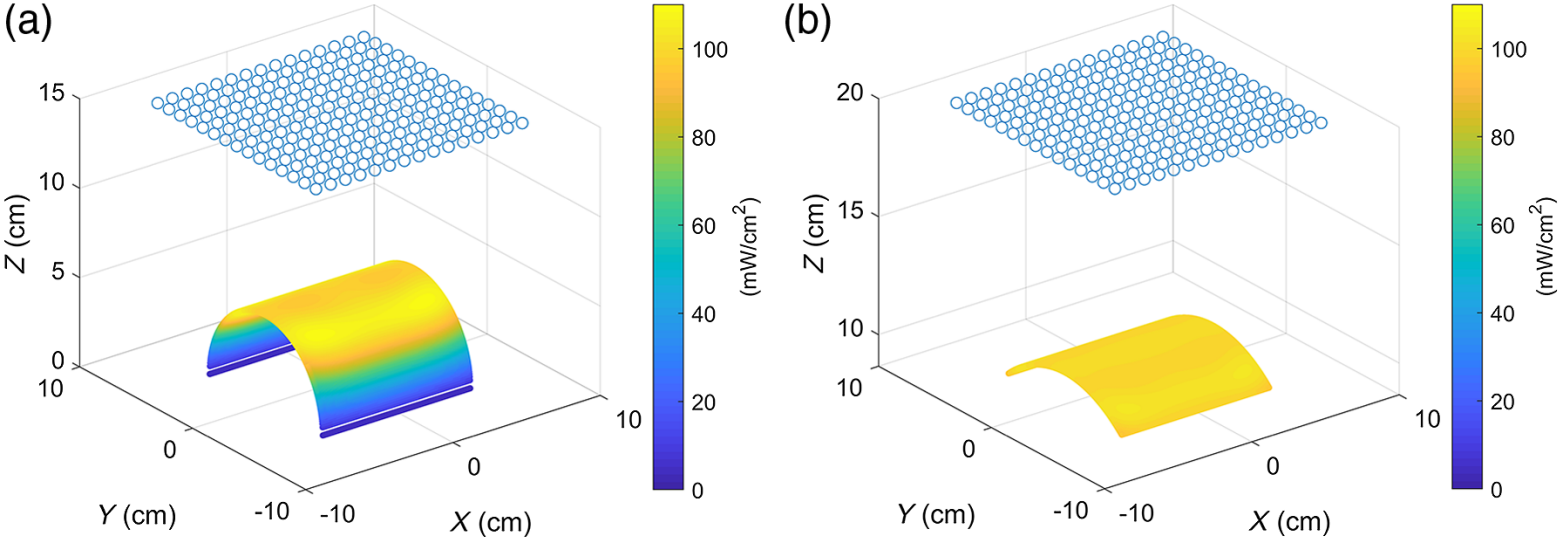
Irradiance distribution on a cylinder surface with a radius of (a) 5 cm and (b) 10 cm.

As indicated in the previous section, meeting the requirements is difficult for the irradiance on the nose and cheek due to their small radii of curvature. When the radius of curvature is only 5 cm [Fig. 9(a)], the angle between the normal vector on the cylindrical surface and the normal vector on the plane of the light source is too large. As such, the light source cannot induce an effective irradiance on the surface, resulting in an effective irradiance occupancy of 75.69% and a coefficient of variance of 25.49%.

However, if the radius of curvature of the cylindrical surface is increased to 10 cm [Fig. 9(b)] and the projected area on the XOY plane remains unchanged, then the effective irradiance occupancy reaches up to 100%, and the coefficient of variance is 1.38%. Thus, the radius of curvature has a considerable influence on the irradiance, which explains the consistently low level of irradiance at the nose and cheek with small radii of curvature.

### 4.2 Number of Clustering Regions

The lesion point cloud was divided into 50 regions in our previous discussion to reduce the difficulty of solving the light intensity matrix of the light source. Figure 3(c) shows that the average area of the clustering region is $∼50\;{\mathrm{mm}}^{2}$. If the lesion point cloud is divided into more categories, then the average area of each clustering region will be smaller and accurate, but the time consumed for solving will be prolonged. Under the condition of 100 mm distance from the light source, when the lesion point cloud is divided into 70 regions, the number of effective points is 1689, the percentage of effective points is 87.15%, and the coefficient of variation is 8.25%. If the lesion point cloud is divided into 100 regions, then the number of effective points is 1696, the percentage of effective points is 87.51%, and the coefficient of variation is 8.11%. Thus, the effective points will only change slightly by increasing the number of clusters, and 50 regions will meet most of the requirements for the irradiance and coefficient of variation.

### 4.3 Tissue Optical Properties

Given that the effective light dose of the inner layer of blood vessels differs from that of the surface, the proposed method achieves only the goal that the optimized light source can produce uniform irradiance on the lesion surface. The effects of tissue optical properties and photosensitizer properties, such as absorption and concentration, and the possibility of photobleaching require further study in the future. Skin optical properties also play important roles in lesion segmentation. Although the segmentation process works well for the Asian head model, it should be optimized for people with other skin tones.

### 4.4 Feasibility for Real-Time Processing

Considering that most patients with port-wine stains are children who are almost impossible to keep still throughout PDT, the capability for real-time processing is of the greatest need for clinical use. As discussed above, the whole procedure includes 3D scanning, lesion segmentation, point cloud clustering, irradiance optimization, and LED current adjustment. The scanning frequency of the 3D scanner can reach 15 Hz (67 ms), and the LED current adjustment can be accomplished within 1 ms using a microcontroller circuit. During PDT, the healthy skin is protected by a black blanket, and the 3D scanner directly scans the lesions to generate point clouds without the need for lesion segmentation. The proposed algorithm is evaluated on our personal computer with 8 GB RAM. The processor is an Intel (R) Core (TM) i5-4210m CPU. The software is implemented with MATLAB 2018a. The time consumed by the proposed method is shown in Table 3. The entire procedure can be completed in <0.3  s. The time consumed for clustering can be further reduced with the development of personal computers. Thus, such a device can be feasibly run at a speed over 5 Hz.

**Table 3:** Time consumption of each step.

Procedure	3D scanning	Point cloud clustering	Irradiance optimization	Current adjustment
Time (ms)	67	200	30	∼1

## 5 Conclusions

An optimized method for an LED array PDT device with a 3D scanner is presented in this paper. This device adjusts its unit current independently according to the relationship between the lesion point cloud and the light source through a 3D scanner. The effective irradiance area proportion and irradiance uniformity of the lesion surface is also improved. This device promotes personalized illumination in PDT for skin diseases and improves efficiency in a single PDT treatment.
